# Trachea repair using an autologous pericardial patch combined with a 3D carbon fiber stent: A case report

**DOI:** 10.3389/fsurg.2022.1086792

**Published:** 2023-01-09

**Authors:** Bin Wang, Zhe Zhang, Yuanwei Guo, Fenglei Yu

**Affiliations:** ^1^ Department of Thoracic Surgery, The Second Xiangya Hospital of Central South University, Changsha, China; ^2^ Hunan Key Laboratory of Early Diagnosis and Precise Treatment of Lung Cancer, The Second Xiangya Hospital of Central South University, Changsha, China; ^3^ Early-Stage Lung Cancer Center, The Second Xiangya Hospital of Central South University, Changsha, China; ^4^ Health Management Center, The Second Xiangya Hospital of Central South University, Changsha, China

**Keywords:** trachea repair, autologous pericardial patch, 3D carbon fiber stent, case report, trachea defect

## Abstract

This study is the first to use an autologous pericardial patch combined with a 3D carbon fiber stent for the plastic repair of a large trachea defect. Radical surgery is the optimal therapy for primary malignant tracheal tumors. Tracheoplasty or repair is required to guarantee trachea integrity and normal ventilation function after tracheal tumor resection. Here, we present a case of plastic repair of the trachea using an autologous pericardial patch and a 3D custom-made carbon fiber stent. A 4 cm trachea defect was successfully repaired after resecting a malignant schwannoma. The postoperative ventilatory function was normal without obvious symptoms of discomfort. Fiberoptic bronchoscopy showed a smooth mucosal surface of the endotracheal wall and patency of the airway. CT scans performed 3 years after surgery showed no recurrence. Therefore, we can conclude that a 3D carbon fiber stent is feasible for abolishing patch floating and preventing tracheal stenosis.

## Introduction

Malignant peripheral nerve sheath tumor (MPNST) or malignant schwannoma is a malignant tumor emanating from the peripheral nerve sheath, which is most often seen in the lower extremities but rarely reported in the trachea (1). Radical resection remains the preferred treatment for MPNST. Tracheoplasty is a complex procedure for repairing a defect formed after the resection of a tumor involving both the trachea and the right main bronchus. Trachea repair with a patch will work as a better and complete solution if we can propose an effective measure to prevent patch floating and tracheal stenosis after patch repair. Herein, we present a unique case of trachea repair using an autologous pericardial patch and a 3D custom-made carbon fiber stent after MPNST resection. The application of the 3D carbon fiber stent is very effective in abolishing pericardial patch floating and preventing tracheal stenosis.

## Case presentation

**Figure 1:**
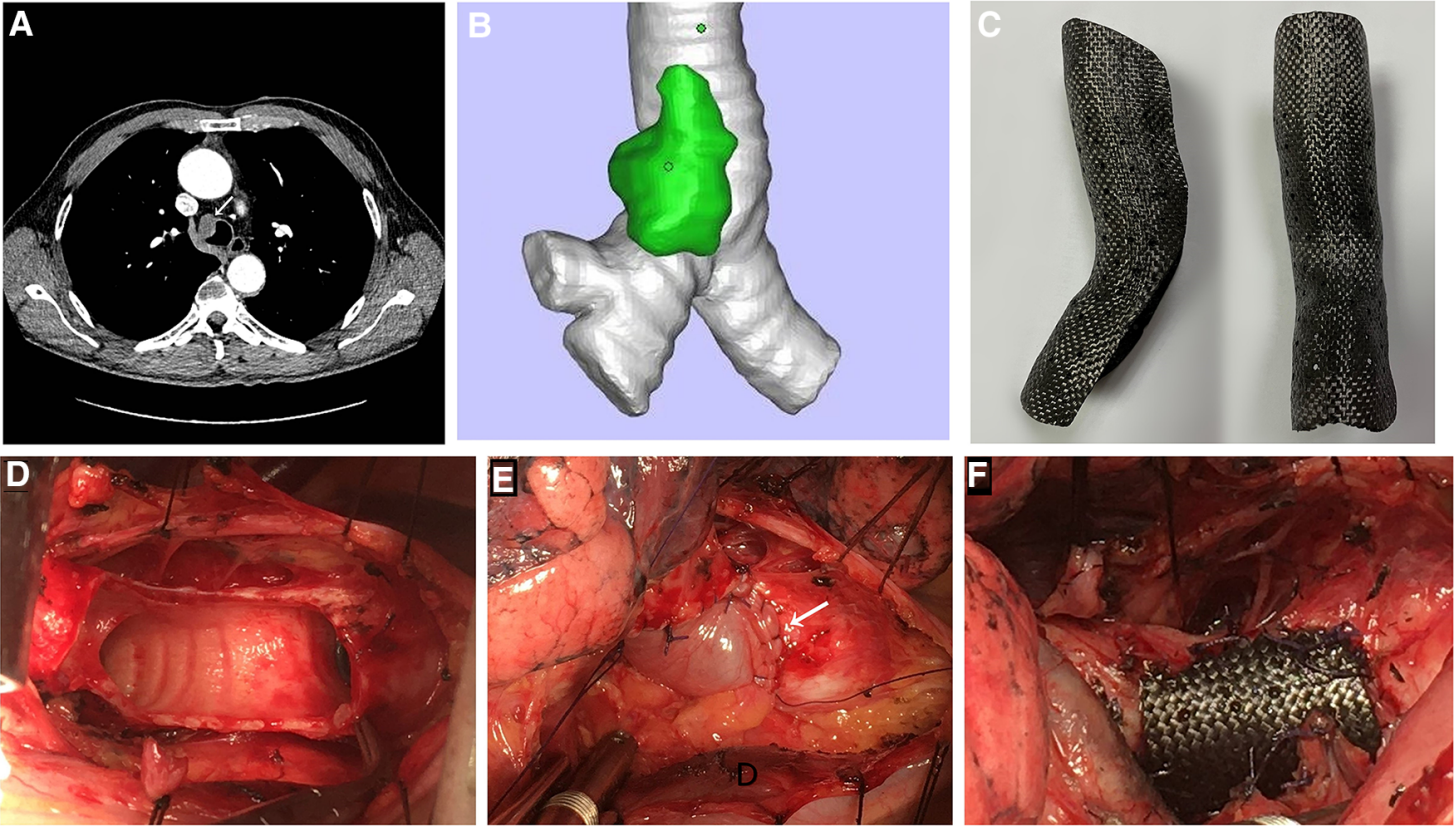
(**A**) Preoperative CT scan of the thorax. The white arrow indicates a tracheal tumor. (**B**) 3D model of the trachea with tumor. (**C**) 3D custom-made carbon fiber stent before tailoring. (**D**) Trachea defect caused by tumor resection. (**E**) Defect repaired by using an autologous pericardial patch. The white arrow indicates a pericardial patch. Airway ventilation will cause a patch float at this point. (**F**) Custom-made carbon fiber stent fixed outside the patch for suspension to abolish a pericardial patch float.

A 55-year-old male presented with a 3.5 cm × 2.8 cm tracheal tumor revealed by computed tomography scan during a medical examination for 2 months but showed no symptoms such as cough or chest tightness. CT showed that the tumor was located in the right lateral wall of both the trachea and the right main bronchus (Figure 1A). A fiberoptic bronchoscopy biopsy of the tumor revealed low-grade malignant schwannoma. Positron emission tomography-computed tomography (PET-CT) showed a 3.5 cm glucose metabolism increased mass in the right lateral wall (maximum standardized uptake value = 5.03) with no lymph node or other metastasis. Preoperative pulmonary function tests showed normal ventilatory and diffuse function (FEV1 was 2.97 L, and FEV1/FEV was 83.8%). Radical tumor resection and trachea repair were planned after a multidisciplinary discussion. Trachea repair in situ using an autologous pericardial patch and a 3D custom-made carbon fiber stent was proposed and approved by the multidisciplinary committee.

**Figure 2:**
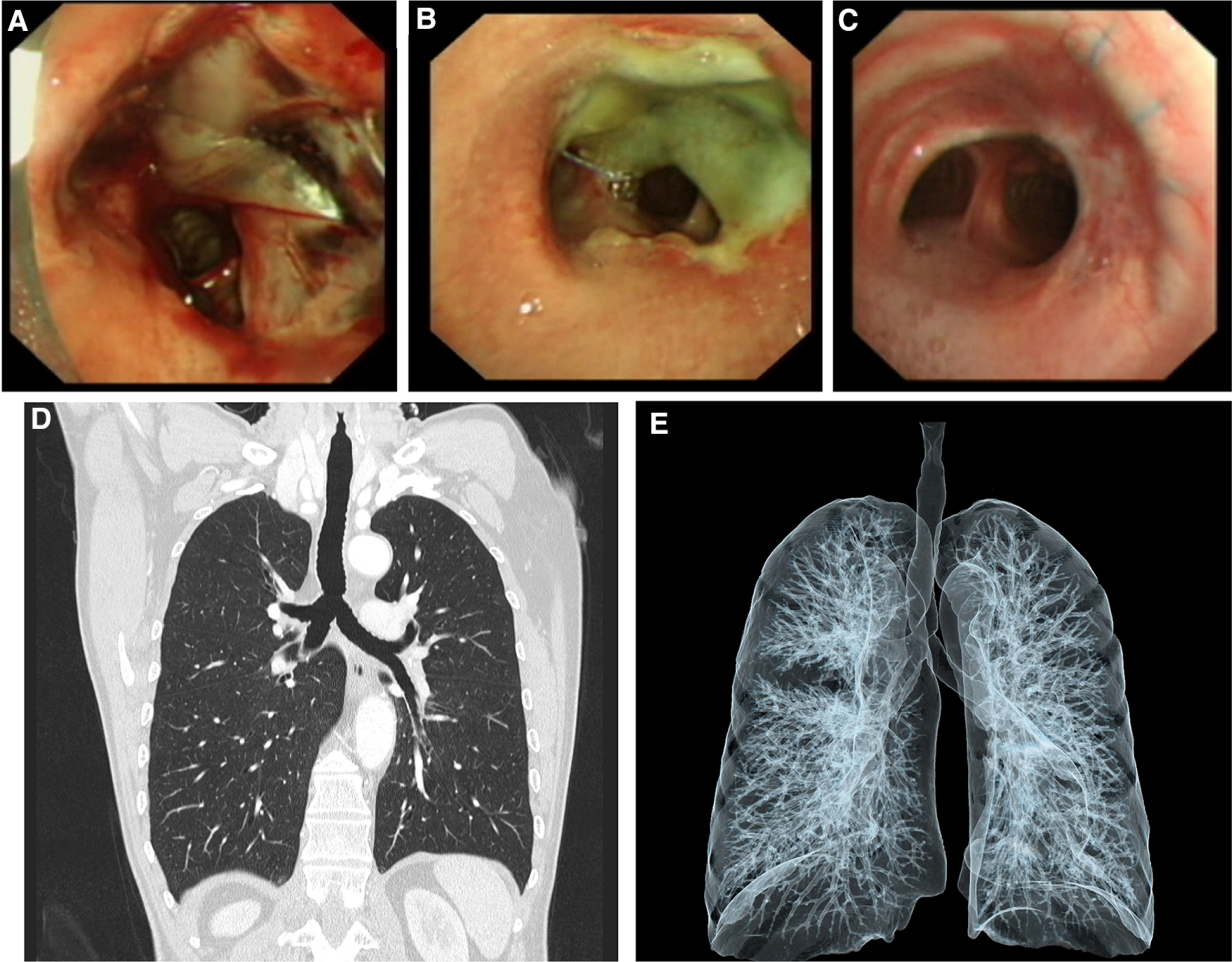
Fiberoptic bronchoscopy on the day of surgery (**A**), 10 days after surgery (**B**), and 6 months later (**C**). Fiberoptic bronchoscopy 6 months later showing a smooth mucosal on the inner surface of the repaired trachea. (**D,E**) Postoperative CT scan and 3D tracheal imaging 3 years later showing no tracheal stenosis or obstruction.

First, a 3D model of the trachea and tumor was created by using MIMICS software (Materialise Magics; Materialise Software, Leuven, Belgium) with CT data (Figure 1B). Then, tumor resection and repair of the tracheal defect were performed in the 3D electron model. Next, a custom-made carbon fiber stent was prepared with the carbon fiber 3D sintering printing technique (Tankang biotech, Changsha, China) (Figure 1C). We performed the tumor resection via right posterolateral thoracotomy until the resection margin was negative. Tumor resection caused a 4 cm length defect in the trachea and the right main bronchus (Figure 1D). A right anterior autologous pericardial patch was first resected and trimmed to repair the defect by a continuous suture with a 3-0 absorbable suture, then the carbon fiber stent was tailored to an appropriate size and fixed outside the patch for suspension to abolish the pericardial patch float (Figures 1E,F), and then, the stent was fixed by an interrupted suture with the 3-0 absorbable suture. After covering the stent with the surrounding soft tissue, we inserted a drainage tube and closed the incision. The operation was very successful, and fiberoptic bronchoscopy showed a clear airway (Figure 2A). The patient had no respiratory symptoms other than a mild cough. After 5 days of anti-infective treatment, the inflammation index returned to normal. Fiberoptic bronchoscopy performed 10 days after the surgery showed a clear airway with a thin layer of inflammatory exudation on the pericardial patch (Figure 2B), and the patient was discharged the next day. The patient was fully free of symptoms 8 weeks after surgery. Fiberoptic bronchoscopy 6 months later showed a smooth mucosal on the inner surface of the repaired trachea (Figure 2C), and the pulmonary function tests showed a normal ventilatory and diffuse function. CT scans showed no tracheal stenosis or obstruction during a 3-year follow-up (Figures 2D,E).

## Discussion

Primary tracheal malignant neoplasm rarely occurs in the upper airway, accounting for less than 0.4% of all newly diagnosed tumors, and it is usually misdiagnosed (2). CT scan, together with trachea bronchoscopy, is commonly applied to diagnose and stage primary tracheal cancer. Squamous cancer is the predominant histological type of tracheal cancer, followed by adenoid cystic carcinoma and neuroendocrine tumors (1). Primary MPNST of the trachea has been rarely reported in the literature and mainly occurs in the cervical and thoracic trachea. The diameter of most of these tumors is less than 2.5 cm when found (3). Surgical resection is the preferred treatment for tracheal MPNST. Endoluminal treatment and radiotherapy can be used as adjuvant treatment options.

Resection of tracheal tumors inevitably leads to trachea interruption or trachea defect. An end-to-end anastomosis is often considered a tracheal interruption that occurs within 4 cm. For large defects of the trachea, trachea repair or tracheoplasty can be considered. A pericardial patch is the best material for repairing trachea defects. It has a smooth surface, tenacity, and is sufficiently air proof. Carter et al. (4) used a bovine pericardial patch to repair an iatrogenic tracheal rupture without flap reinforcement, which is the first such report of a successful bovine patch repair without accessory autologous tissue reinforcement. However, most surgeons tend to use an autologous pericardial patch for tracheoplasty or repair, especially for congenital tracheal stenosis and tracheomalacia. Fiore et al. (5) reported the case of six patients who underwent tracheoplasty with an autologous pericardial patch. However, only two of the six had a good surgical outcome. One patient required reoperation for a tracheostomy, and three patients died in the early or late postoperative period. Aria's team performed tracheoplasty in 21 patients using an autologous pericardial patch and strips of costal cartilage. Of these, only two patients had complications of mediastinitis and patch dehiscence. Three died in the late postoperative period with the following conditions: one died of sepsis, one had patch dehiscence, and another suffered from an erosion of the tracheal stent and consequently intractable bleeding. A total of 12 (67%) patients were free of symptoms during follow-up. The surgical outcomes using the autologous pericardial patch and costal cartilage were significantly better than those of other reports.

In our case, because of the defect involving both the trachea and the right main bronchus, direct shaping without a patch would have complicated the operation. Patch floating would have occurred if we had performed trachea repair with an autologous pericardial patch alone because of the large-sized defect, and tracheal stenosis and obstruction can easily occur in the late postoperative period. Therefore, combining the results of Aria's study with our own experience with 3D carbon fiber reconstruction implants (6–8), we designed a customized 3D carbon fiber stent outside the pericardial patch. The floating of the patch and tracheal stenosis were avoided by applying stent traction. The follow-up results of 3 years post-surgery also confirmed our hypothesis. However, we faced some limitations during the treatment of this patient. The time required to perform 3D carbon fiber stent customization was approximately 10 days, and the cost of customization was too high, which are unacceptable conditions for emergency surgery patients or those who are financially disadvantaged. Here, it is worth emphasizing that the implant failure of a carbon fiber stent due to displacement may result in tracheal leakage or stenosis. Therefore, a fully experienced thoracic surgeon is required for performing the operation. We are unaware of the size limit for the carbon fiber stent, but external stenting within 4–6 cm should be safe.

In conclusion, this is the first report of a successful trachea repair using an autologous pericardial patch combined with a 3D carbon fiber stent. This was a novel individual treatment procedure that not only produced a satisfactory result but also provided new ideas and a rich experience for us to further explore the clinical application of artificial trachea.

## Patient perspective

The patient completed a questionnaire 3 years after the operation, entitled The European Organisation for Research and Treatment of Cancer Quality of Life Questionnaire Core 30 (EORTC QLQ-C30). The EORTC QLQ-C30 score 3 years after the surgery showed that all the functional domain scores were above 100%, the symptom domain score was 0%, and the overall health status score was 91.7%. The patient reported that they only had a mild cough and wound pain in the early days after surgery and that there was a gradual improvement in their condition thereafter.

## Data Availability

The original contributions presented in the study are included in the article/Supplementary Material; further inquiries can be directed to the corresponding author.
